# Relationship between lay and expert perceptions of COVID-19 vaccine development timelines in Canada and USA

**DOI:** 10.1371/journal.pone.0262740

**Published:** 2022-02-15

**Authors:** Patrick Bodilly Kane, Hannah Moyer, Amanda MacPherson, Jesse Papenburg, Brian J. Ward, Stephen B. Broomell, Jonathan Kimmleman

**Affiliations:** 1 STREAM Research Group, Division of Ethics and Policy, School of Population and Global Health, McGill University, Montreal, QC, Canada; 2 Division of Pediatric Infectious Diseases, Dept. of Pediatrics, The Montreal Children’s Hospital, McGill University Health Centre, Montreal, Quebec, Canada; 3 Research Institute of the McGill University Health Centre, Montréal, Québec, Canada; 4 Department of Social and Decision Sciences, Carnegie Mellon University, Pittsburgh, PA, United States of America; St John’s University, UNITED KINGDOM

## Abstract

**Objectives:**

Compare lay expectations of medical development to those of experts in the context of SARS-CoV-2 vaccine development.

**Methods:**

A short online survey of experts and lay people measuring when participants believe important vaccine milestones would occur and how likely potential setbacks were. Samples of US and Canadian lay people recruited through Qualtrics. The expert sample was created through a contact network in vaccine development and supplemented with corresponding authors of recent scholarly review articles on vaccine development.

**Results:**

In aggregate, lay people gave responses that were within 3 months of experts, tending to be later than experts for early milestones and earlier for later milestones. Median lay best estimates for when a vaccine would be available to the public were 08/2021 and 09/2021 for the US and Canadian samples, compared with 09-10/2021 for the experts. However, many individual lay responses showed more substantial disagreement with expert opinions, with 54% of lay best estimates of when a vaccine would be available to the public being before the median expert soonest estimate or after the median expert latest estimate. Lay people were much more pessimistic about vaccine development encountering setbacks than experts (median probability 59% of boxed warning compared with only 30% for experts). Misalignment between layperson and expert expectations was not explained by any demographic variables collected in our survey.

**Conclusion:**

Median lay expectations were generally similar to experts. At the individual level, however, lay people showed substantial variation with many believing milestones would occur much sooner than experts. Lay people were in general much more pessimistic about the prospect of setbacks than were experts.

## Introduction

The lay public follows medical developments through news stories, social media and/or charitable organizations. For example, many patients and their family members routinely scan media reports to learn about emerging treatment paradigms.

Content analyses suggest that such media reports often overstate the promise of novel treatments, [1–3] leading to concerns about unrealistic expectations among patients. For example, news articles about new cancer treatments often use superlatives, even though treatments have small effects on surrogate measures of disease [4]. Numerous commentators warn that hyperbolic news accounts may distort patient decision-making; they also warn that expectations engendered by such coverage can lead to disappointment and disillusionment as promised advances fail to materialize on projected outcomes [5–7]. Embedded within such criticism, however, is an assumption that publics are passive and credulous consumers of media coverage of new medical treatments, or that misapprehensions about new medical technologies are not adequately buffered by other information sources.

The extent to which public expectations surrounding medical innovation align with consensus views of experts has not been well characterized in medicine. In the only previous study we are aware of, Kane et al. [8] surveyed a sample of patients and experts in Parkinson’s disease regarding expectations for the attainment of major research milestones in the coming decade. Although patients generally expected major advances on nearer timelines than experts, these misalignments were small in magnitude. These findings suggest that many patients with serious chronic illnesses are critical consumers of information about medical advances.

SARS-CoV-2 vaccine development generated an enormous amount of media coverage, providing a natural opportunity to investigate the relationship between public and expert expectations in a similar setting of substantial clinical need. Lay and expert alignment may be especially important in the context of vaccine deployment, given a long history of research suggesting lay people generally view vaccines as much riskier than experts [9]. Vaccine vaccne hesitancy is well documented [10–12] and has become an important policy issue as vaccination efforts in various jurisdictions stalls. Medical reversals, as when public health agencies change policies in response to emerging science or unexpected setbacks, may be perceived as undermining the credibility and political independence of medical experts.

While the relationship between public and expert expectations surrounding new medical technologies has implications for numerous policies, it has particular relevance in public health crises, where maintaining credibility and confidence of various publics is paramount. Understanding lay persons’ expectations about vaccines compared to experts when they were still in development may help to shed light on current challenges with COVID-19 vaccine hesitancy. Additionally, given the variable impact of and response to COVID-19 among different demographic groups, understanding where misalignments are most concentrated among publics, and why these misalignments occur, may be key to devising better communication and public engagement strategies [13].

In what follows, we report a comparison between the expectations of US and Canadian lay people and the previously published predictions of group of experts regarding timelines and potential setbacks for SARS-CoV-2 vaccine development as of the summer of 2020 [14]. This comparison allows us to explore to what extent the beliefs of experts at the time were being successfully communicated to lay people, and possibly whether there is room for improvement in the communication process.

## Methods

### Sample

We created a sample of vaccine experts in academia, industry or in public health using the contact network of one of the authors (BJW, an expert in vaccine development). We sought diversity across gender, age, and employment sector. Personal contacts were sought to enhance response rates from experts who are currently under extraordinarily high demand. This contact list was supplemented with corresponding authors identified by a literature search for review articles on vaccine development in major journals. Details of our expert sample are reported in the Supplement and elsewhere [14].

Two samples of laypersons (US and Canada) were recruited through Qualtrics (Provo, UT, United States). Samples were generated to be representative of their respective populations based on available census data for age and gender. Qualtrics was unable to sample for representative educational attainment. All participants provided informed consent electronically; our protocol received approval from McGill IRB.

### Survey

Given the fast-moving nature of the COVID-19 pandemic (and the fact that vaccinologists were besieged by requests for their expertise early in the pandemic), we designed a simple survey that could be implemented quickly and completed by both experts and lay persons with an economy of time and effort. We sought forecasts from experts for three temporally ordered milestones in vaccine development: 1) a field study enrolling at least 5000 participants reporting results, 2) a vaccine being available to those at highest risk from the virus in the US and/or Canada, and 3) a vaccine being available to the general public in the US and/or Canada. We began lay surveys by asking how far participants thought a vaccine had progressed through the research process. For each milestone that a participant indicated had not yet occurred, we elicited a best estimate for when the milestones would be achieved, and their predictions for soonest and latest occurrence. We also collected estimates of the probability of two setback events: 1) the first vaccine widely deployed in the US and/or Canada receiving a boxed warning from the FDA (the FDA issues boxed warnings when a safety issue is detected after a vaccine has been approved) and 2) the first large field trial in the US and/or Canada reporting a null or negative result on its efficacy endpoint. Lay surveys were identical to expert surveys except for several additional questions about vaccine development knowledge and demographic questions, and text additions to explain on technical terms (see Supplemental Material).

Gender, age, education, ethnicity, political stance on a 7-point Likert scale from strong left to strong right, and political affiliation were also collected from laypersons. Finally, we assessed individuals’ confidence in scientific efforts involving COVID-19 using several questions with 5-point Likert scales. Due to a lack of internal consistency across question responses, only a single question from this assessment was used in the analysis: whether individuals agreed with the statement “The increased urgency associated with COVID-19 research will lead to more errors in the research and development process.” as decided in advance according to our preregistered analysis plan (see the Supplemental Materials for details of the scale and its reliability).

The expert survey was administered from 6/23/20 to 7/5/20; the US lay survey was administered from 6/30/20 to 7/10/20; the Canadian lay survey was from 7/1/20 to 7/21/20. At the time 3 vaccine candidates had reach Phase 3 testing, another 8 were in Phase 2 testing and 11 were in Phase 1 testing.

### Data cleaning

See Supplemental Materials for description of our data cleaning which followed our protocol in removing inconsistent responses (https://osf.io/faz9q). Details on the number of responses removed can be found in S3 Table in S1 Appendix.

### Analysis

We calculated descriptive statistics for (a) proportion of participants indicating that each milestone had already occurred, (b) the median forecast for best estimates, soonest estimates and latest estimates of participants indicating that each milestone had not yet occurred, and (c) the median probability for each setback question.

We also preformed statistical tests comparing responses across the three samples (expert, US lay and Canadian lay) for the timeline and setback questions. Experts were treated as one group regardless of nationality and all participants were asked about events occurring “in the US and/or Canada” due to both the limited number of expert participants and the idea that experts are generally paying attention to international developments in a way lay people might now be. Of the participants who correctly indicated that milestones had not yet occurred, we tested for differences across each of the three pairs of samples (expert versus US lay, expert versus Canadian lay and US lay vs Canadian lay) in their median best estimate for each milestone using a bootstrapped t-test (treating the expert median best estimate as a constant). These tests were adjusted for multiplicity such that the critical value was 0.05/24≈0.002 (this multiplicity adjustment includes tests for differences in the range between soonest and latest estimates reported in the Supplemental Materials). Likewise, we tested for differences across each pair of samples in the median probabilities for each setback using a bootstrapped t-test (treating the expert median best estimate as a constant).

We deviated from our protocol to perform one exploratory analysis of the proportion of lay people whose best estimates fell outside the bounds of median expert soonest and latest estimates. The lay responses were placed into one of three categories: before the median expert soonest estimate (this includes individuals who believed a milestone had already occurred); after the median expert latest estimate; and between the median expert soonest and latest estimate. We tested whether the distribution of these categories was different between the US and Canadian samples with Chi-squared tests of independence.

Per our protocol we also performed an exploratory analysis testing which if any demographic variables predicted how far an individual’s expectations were from the median expert expectation to see if any groups harbored notable levels of optimism or pessimism relative to expert forecasts. For each milestone best estimate and setback question, we subtracted the expert median from each lay response and performed multivariate linear regression of this deviation on the demographic variables we had collected (histograms of these deviation variables can be found in S5 and S6 Figs in S1 Appendix). Specifically, we regressed on all of the demographic variables we collected: educational attainment, gender, age, ethnicity, political affiliation. We also controlled for how far individuals believed a vaccine had already progressed through the approval process (a categorial variable with levels for different stages reached by the most advanced vaccine) and agreement with the statement “The increased urgency associated with COVID-19 research will lead to more errors in the research and development process” on a 5-point Likert scale (information on these two variables can be found in S5 Table in S1 Appendix). Finally, we included a nationality interaction on all of these terms to allow for the possibility that coefficient values differed among US and Canadian samples (e.g. because of a more politically polarized media environment in one of the countries). Statistical significance was determined with the standard alpha of 0.05. Unless otherwise noted, we did not adjust for multiple hypothesis testing, as tests were exploratory.

All analysis was performed in R Version 4.0.2 (R Foundation for Statistical Computing, Vienna, Austria).

## Results

### Samples

The expert sample consisted of 28 participants out of 72 experts solicited for participation (details published elsewhere) [14]. According to past work on expert forecasting, this was sufficient sample size to achieve a stable estimate of expert consensus [15, 16]. The US lay sample consisted of 1015 participants. The median age was 42; 40% of the sample identified as Democrats, 32% as Republicans and 26% as Independents. The Canadian sample consisted of 1049 participants. The median age was 44; 38% identified as Liberal, 27% Conservative, 13% NDP, 5% Green, 0.5% Bloc Quebecois, 6% Independent and 7% other. For further demographic information, see Table 1.

**Table 1:** Demographic breakdown of lay participants.

Gender	US Sample	Canadian Sample
Female	50%	53%
Male	49%	46%
Other Answer	1%	1%
**Educational Attainment**		
High School	49%	38%
Undergraduate	29%	36%
Graduate	18%	13%
Other Answer	3%	11%
**Ethnicity**		
Asian	5%	21%
Black	14%	3%
Latinx	6%	1%
White	69%	66%
Other Answer	6%	8%
**Political Stance**		
Far Right	12%	4%
Right	14%	11%
Lean Right	12%	17%
Center	39%	39%
Lean Left	8%	14%
Left	9%	10%
Far Left	7%	3%
**Political Affiliation**		
Democrat	40%	-
Republican	26%	-
Independent	32%	6%
Liberal	-	38%
Conservative	-	27%
NDP	-	13%
Green	-	5%
Bloc Quebecois	-	1%

### Milestone predictions

**Fig 1:**
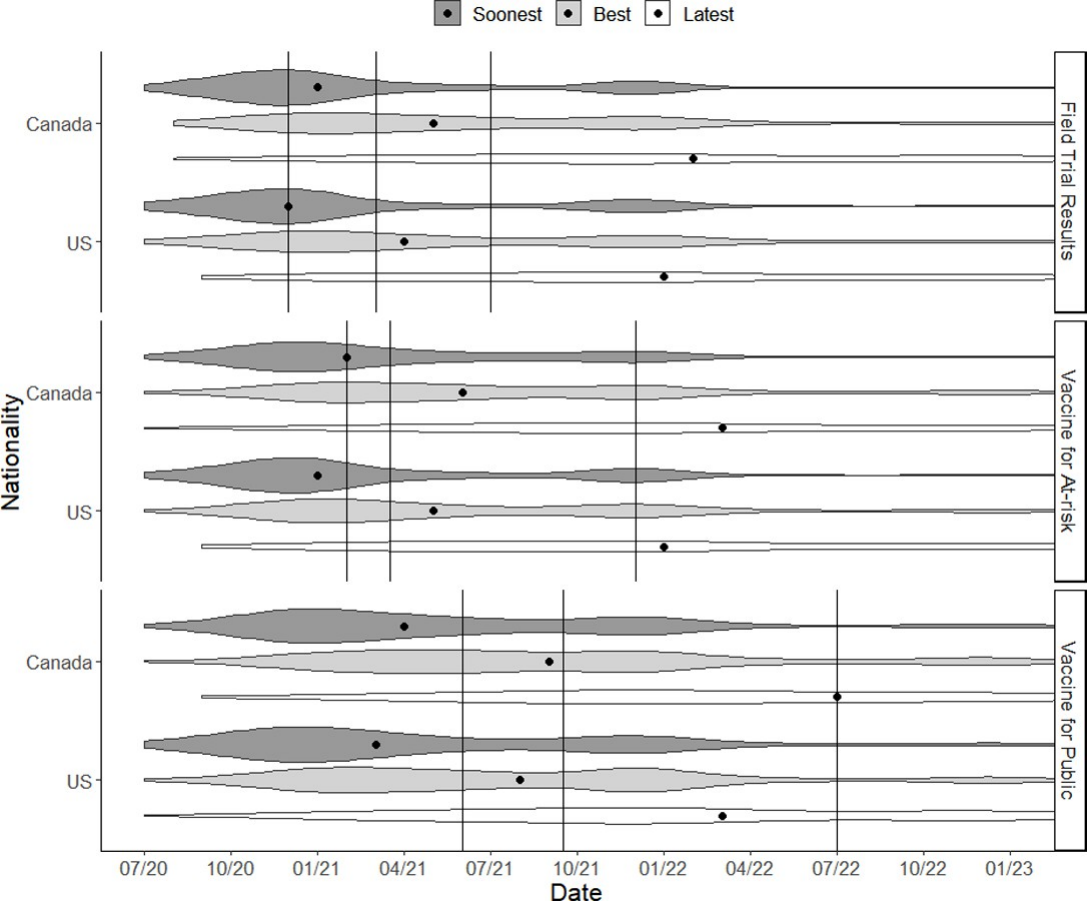
Violin plots of the best estimate, soonest estimate and latest estimate for each milestone and lay sample. Black dots represent the lay median for each plotted distribution. Vertical lines represent the median expert soonest, best guess and latest guess respectively.

Fig 1 shows the distributions of lay responses for each milestone. Median best, soonest, and latest lay estimates were very close to those of experts while the interquartile range of lay responses was wider than that of experts. Despite differences amounting to small effect sizes, bootstrapped t-tests for differences in medians reveal that both the median US and median Canadian best estimate for when a field study would report results were significantly later than the median expert best estimate. Likewise, bootstrapped t-tests found the Canadian median best estimate for when the vaccine would be available to those most at risk was significantly later than both the US and expert median best estimate. The largest of these significant differences was two and half months and the smallest was one month. There were no significant differences in projected timelines for vaccine availability for the general public (Table 2).

**Table 2:** Differences in median estimates for the three milestones and the two setback questions for each sample. Positive values indicate the first listed sample making an earlier forecast or giving a higher probability respectively. The p-value section refers to the p-value in a non-parametric bootstrap comparison of the medians for each pair of samples. The critical values used in this analysis were Bonferroni corrected (0.05/24 = 0.002) because of the multiple hypotheses tested for in these data (including several hypotheses discussed in the Supplemental Material). Asterisk indicated statistical significance.

	Difference in Median Estimates			P-value		
Question	Expert vs US	Expert vs Canada	US vs Canada	Expert vs US	Expert vs Canada	US vs Canada
**Field Study Results**	1 month	2 months	1 month	<0.001*	<0.001*	0.055
**Vaccine for At-Risk**	1.5 months	2.5 months	1 month	0.024	<0.001*	<0.001*
**Vaccine for Public**	-1.5 months	-0.5 months	1 month	0.007	0.116	0.032
**Boxed Warning**	29%	29%	0%	<0.001*	<0.001*	0.772
**Null Outcome**	15%	15%	0%	<0.001*	<0.001*	0.930

Table 3 contains the proportion of lay best estimate responses falling outside the median expert soonest and latest estimates. For each milestone, more than 50% of lay best estimates fell outside the bounds of the median expert soonest and latest estimates. These out of bound responses were biased towards later-term occurrence for the “field study reporting results” milestone but biased towards nearer term occurrence for the other two milestones. For example, in the case of the vaccine being available to the general public, almost half of lay best estimates were before the median expert soonest estimate while 17% were after the median expert latest estimates. For each milestone the distribution across these categories differed such that a greater proportion of US responses occurred before the median expert soonest guess in comparison to the Canadian sample (p<0.001 for the phase 3 trial milestone, p<0.001 for the vaccine being available to those most at risk milestone, and p<0.001 for the vaccine being available to the public milestone).

**Table 3:** Relationship between lay best estimates and expert soonest/latest estimate boundaries. The Before Soonest column includes responses where participants indicated they thought the event had already occurred.

US	Before Soonest	Within Bounds	After Latest
Field Study Results	28% (224)	39% (315)	33% (261)
Vaccine for At-Risk	36% (272)	45% (341)	18% (137)
Vaccine for Public	43% (315)	43% (312)	15% (107)
**Canada**			
Field Study Results	17% (156)	47% (422)	36% (322)
Vaccine for At-Risk	25% (219)	53% (469)	22% (192)
Vaccine for Public	33% (286)	49% (422)	18% (159)
**Overall**			
Field Study Results	22% (380)	43% (737)	34% (583)
Vaccine for At-Risk	30% (491)	50% (810)	20% (329)
Vaccine for Public	38% (601)	46% (734)	17% (266)

### Perceived chances of setbacks

**Fig 2:**
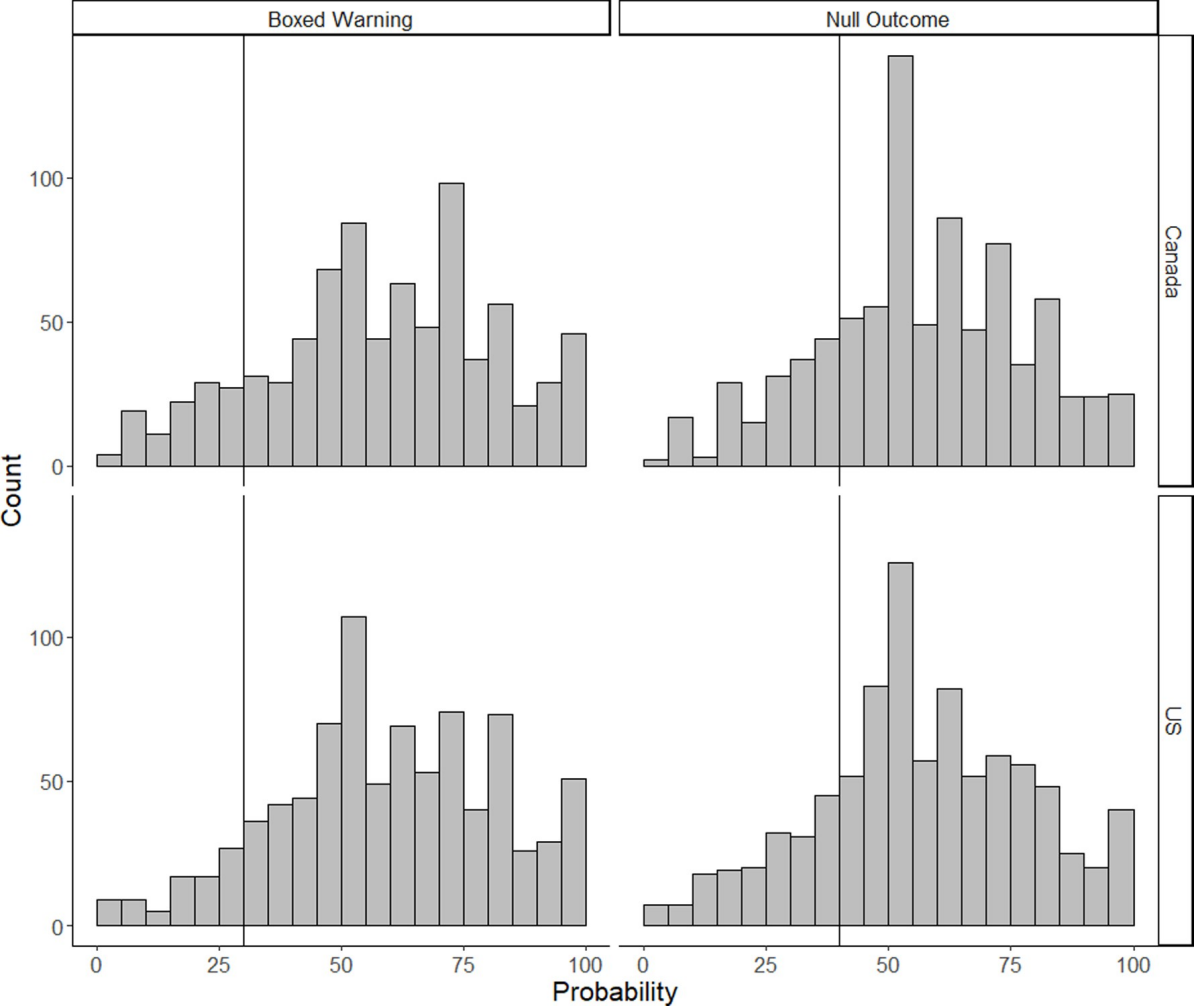
Histograms of predicted probabilities for the potential setbacks for each lay sample. The vertical line represents the median expert probability.

Fig 2 presents histograms for the two setback questions: the probability of an approved vaccine receiving a boxed warning and the probability of the first large trial returning a null outcome. For the US lay sample, the median probabilities were 59% and 55%, respectively. For the Canadian lay sample, the median probabilities were also 59% and 55%, respectively. By comparison the expert sample medians were 30% and 40%. Bootstrapped t-tests found that for both questions the expert sample medians significantly differed from the US lay and Canadian lay sample medians (see Table 2).

### Individual differences in milestone predictions

The results of nested model comparisons testing whether each demographic variable is a significant predictor along with the overall R^2^ for each regression are reported in Table 4. The full list of coefficients for each regression are reported in the S6 and S7 Tables in S1 Appendix. Even significant predictors of milestone estimates did not account for very much of the variation in the data (e.g. adjusted R^2^ did not exceed 0.10 for any regressions). For the setback forecasts, the only significant predictor for both was how much participants agreed with the statement that “The increased urgency associated with COVID-19 research will lead to more errors in the research and development process,” with higher agreement correlating with higher probabilities of setbacks (b = 6.57, p<0.001 for the boxed warning setback question; b = 6.69, p<0.001 for probability of null outcome question). Gender was also a significant predictor for the boxed warning setback question with women giving higher probabilities than men (b = 4.84, p = 0.01). The nationality interaction was not significant in any regressions.

**Table 4:** Results of F-tests for nested model comparisons testing whether each individual predictor significantly improved the fit of the models, along with the effect sizes on these tests (η^2^). DFs refers to degrees of freedom. Critical values were not adjusted for multiple hypothesis testing. R^2^ refers to the variance explained by the full regression.

	Variable	F	DFs	P-value	η^2^
**Best Estimate of Field Study Result (R**^**2**^ **= 0.03)**	Education	2.27	(6, 1389)	0.03*	0.0098
	Gender	1.2	(4, 1389)	0.3	0.0034
	Age	0.94	(2, 1389)	0.4	0.0013
	Ethnicity	2.05	(8, 1389)	0.04*	0.012
	Political	1.24	(2, 1389)	0.28	0.0018
	Belief about Stage of Approval Process	4.71	(2, 1389)	<0.01*	0.013
	Urgency of Research Causes Errors	2.36	(2, 1389)	0.09	0.0034
	Nationality Interaction	1.03	(14, 1389)	0.41	0.01
**Best Estimate of Vaccine for At-Risk (R**^**2**^ **= 0.03)**	Education	1.84	(6, 1452)	0.09	0.0075
	Gender	1.63	(4, 1452)	0.16	0.0045
	Age	0.92	(2, 1452)	0.4	0.0013
	Ethnicity	1.24	(8, 1452)	0.27	0.0068
	Political	0.28	(2, 1452)	0.74	0.004
	Belief about Stage of Approval Process	4.73	(2, 1452)	<0.01*	0.013
	Urgency of Research Causes Errors	2.03	(2, 1452)	0.13	0.0028
	Nationality Interaction	1.58	(14, 1452)	0.07	0.015
**Best Estimate of Vaccine for Public (R**^**2**^ **= 0.09)**	Education	1.1	(6, 1429)	0.37	0.0046
	Gender	1.85	(4, 1429)	0.11	0.0052
	Age	0.26	(2, 1429)	0.77	0.0004
	Ethnicity	1.35	(8, 1429)	0.21	0.0075
	Political	0.52	(2, 1429)	0.59	0.0007
	Belief about Stage of Approval Process	4.98	(2, 1429)	<0.01*	0.014
	Urgency of Research Causes Errors	5.12	(2, 1429)	<0.01*	0.0071
	Nationality Interaction	1.59	(14, 1429)	0.07	0.015
**Probability of Black Box (R**^**2**^ **= 0.09)**	Education	1.55	(6, 1542)	0.16	0.006
	Gender	4.84	(4, 1542)	<0.01*	0.012
	Age	0.05	(2, 1542)	0.94	0.0001
	Ethnicity	0.51	(8, 1542)	0.84	0.0027
	Political	2.17	(2, 1542)	0.11	0.0028
	Belief about Stage of Approval Process	1.14	(2, 1542)	0.34	0.003
	Urgency of Research Causes Errors	68.86	(2, 1542)	<0.01*	0.082
	Nationality Interaction	1.31	(14, 1542)	0.17	0.012
**Probability of Null Outcome (R**^**2**^ **= 0.10)**	Education	0.95	(6, 1542)	0.46	0.0035
	Gender	0.98	(4, 1542)	0.42	0.0024
	Age	0.87	(2, 1542)	0.42	0.0011
	Ethnicity	0.91	(8, 1542)	0.51	0.0045
	Political	0.95	(2, 1542)	0.39	0.0012
	Belief about Stage of Approval Process	0.75	(2, 1542)	0.56	0.0019
	Urgency of Research Causes Errors	83.81	(2, 1542)	<0.01*	0.094
	Nationality Interaction	0.92	(14, 1542)	0.54	0.0079

## Discussion

Our results suggest that, in the aggregate, US and Canadian lay expectations about when a vaccine would be available to the public taken during the summer of 2020 were largely consistent with those of experts collected at the same time. If we consider the expert judgments as the best information available at the time, this reflects a “wisdom of the crowd” effect where on average, lay expectations are surprisingly close to the expert expectations [17]. For the other two milestone events, median lay responses were significantly different from those of experts for at least one group. However, these statistically significant differences were small in magnitude when compared with the time lines both experts and lay people thought events would unfold along. All significant differences were approximately 2 months while the soonest any of the samples thought an event would occur was on average at least 9 months off. Given positive results from initial large trials, vaccine approvals in December 2020, [18, 19] vaccine distribution to the health care workers in the US in mid-December, [20] and vaccine dissemination to the general public in US and Canada in the late spring, [21, 22] it would appear that at least in aggregate both lay people and experts were overly pessimistic about how long the vaccine development and deployment process would take. It is also worth noting that, for the lay samples, the median Canadian best estimates tended to be behind the US best estimates. This difference was born out by the actual Canadian deployment of vaccines to the general public being approximately two months behind the US vaccine rollout. Overall, the concordance between aggregate lay opinions and expert forecasts suggests that communication about timelines for this particular medical advance, a SARS-CoV-2 vaccine, was successful in relaying expert beliefs to lay people.

Despite median forecasts being similar, a large portion of individual lay people provided estimates that were very discordant with expert forecasts. More than half of ‘best estimate’ forecasts across the US and Canadian lay-persons fell outside median soonest-latest ranges of aggregate expert forecasts. In general, such misaligned expectations tended towards expectation of nearer term accomplishments for laypersons, though there were still a substantial number of extremely pessimistic laypersons.

In contrast to the milestone questions, laypersons showed different and more pessimistic expectations regarding setbacks. Across both samples in a *post hoc* analysis, there was a positive correlation between lay best estimates for when a vaccine would be available to the public and probabilities of a boxed warning on an approved vaccine (*r* = 0.14, p<0.001) indicating that those who thought a vaccine would take longer also thought it would be less safe. Concerns about vaccine safety have been well documented in dealing with existing vaccines for other diseases and have also been documented in public polling [23, 24] and in studies of the public’s intent to take a vaccine where, even among those who intended to get a vaccine, concerns about safety and efficacy were high [25]. Concerns about vaccine safety may be especially high given label changes to the AstraZeneca vaccine [26].

That 54% of lay best estimates for when a vaccine would be publicly available fell outside expert boundaries suggests that there were some issues in the communication of expert knowledge to lay people (or perhaps merely a disbelief in what was communicated). This is not unique to SARS-CoV-2 vaccines; previous research has found that many members of the public harbor inaccurate beliefs about other vaccine research efforts (e.g. believing that a vaccine for HIV exists [27]). However, the highly variable range of opinions, and the simultaneous presence of optimism about timelines and pessimism about risks suggests that a simple account of overly optimistic media reports creating unrealistic expectations is unlikely to be true. Our regression analysis did not suggest any sociodemographic groups were particularly prone to deviating from expert opinions. This is consistent with other work that found that public health behavior with respect to COVID-19 is not well predicted by demographic variables [28].

Our study has several limitations. First, many lay people provided inconsistent responses that indicated poor understanding or disengagement with survey questions. This was a larger issue in the US sample, but responses were overall similar between the two samples. However, the proportion of inconsistent responses is typical for such research [29, 30]. Second, studies of near- term forecasting present analytical challenges, because forecasts are bounded by the present. Lay estimates showed high variation. When such variation is compressed due to the proximity a near-term boundary (as it likely was for our first two milestones), average estimates drift towards later time periods. This may partly explain why lay median estimates were more pessimistic relative to experts on the first two milestones, but not the third. It may also explain why there tended to be more layperson best estimates occurring after the latest expert estimate than before the soonest expert estimate for the earliest milestone relative to the other two. Third, our surveys were conducted in July 2020 and multiple vaccines have reported positive outcomes in large trials since then. As a result, our report should not be used to understand current COVID-19 vaccine perceptions, but our finding of individual level discordance may suggest that some lay people continue to hold very different beliefs than experts. Finally, our study focuses on a very specific scenario–perceptions of the development of vaccines during a global pandemic. Our results are likely impacted both by the highly publicized nature of pandemic intervention development and the fact that the interventions being developed were vaccines and may not generalize to other kinds of interventions. Similarly, our surveys focused on timelines for milestone attainment. Different results might have been obtained had we queried on, the magnitude of public health impact for various milestones.

The challenges to generalization of results beyond our samples bear special attention. First, while our samples were generated in a representational manner regarding a few demographic characteristics, we cannot be certain the samples are perfectly representative of the broader lay populations. For example, this survey was conducted online which may have prevented some less technologically savvy people from participating. Likewise, while we treated lay responses from the US and Canada as two different samples, we did not do the same with experts. This reflected our limited sample size, and the fact that experts are generally in international circulation. It may be that larger samples of experts would have revealed differences in opinion between those in the US and Canada. Additionally, because our expert sample is qualitatively different from our public samples with regard to sample size and demographic variables, we treat the expert sample’s median predictions as a fixed value and compare the distribution of public responses to this value. The limitation of this comparison is that distributional characteristics of the expert judgments are not used in our analysis because the variance of expert opinion cannot be obtained in a way that is comparable to the variance of public opinions. Nevertheless, our work provides a snapshot of how lay people and experts interpreted the information available to them in the summer of 2020 when only three candidate vaccines had entered Phase 3 trials and no vaccine had received Emergency Use Authorization. Finally, our study did not attempt to connect layperson expectations with particular media reports that they might have encountered. However, vaccine development had received widespread coverage in almost every form of mainstream media and was the topic of numerous government press conferences in the US. Though experts in our sample were more pessimistic than media coverage (which cited an early 2021 date for when vaccines would be available to the public [31]), in this case projections in the media turned out to be more accurate.

Our study suggests that, in aggregate, layperson expectations regarding vaccine development timelines did not appear to be misaligned with those of experts. Nevertheless, the aggregate similarity in forecasts between lay people and experts belies the fact that many lay people’s beliefs about vaccine milestone fell outside the bounds of what experts believed plausible. This suggests that lay people may have very different interpretations of optimistic information they receive through news reports, social media and other sources. Such discordant expectations may continue to dog public health efforts as authorities continue to try to boost uptake of deployed vaccines. The discrepancy between lay and expert expectations about vaccine setbacks may imply they differences from experts in perceptions of the safety and reliability of the development process’s output, which could help to explain current issues in vaccine uptake. More broadly, our work suggests a need for further study of variables that can explain misalignment of layperson and expert expectations regarding the value and impact of medical advances.

## Supporting information

S1 Appendix Appendix containing additional information on previously published data, cleaning of data and additional analyses. (DOCX)

S1 File Zipped archive of CSV files containing lay survey data. (ZIP)
