# Supplementary material for: Relationship between lay and expert perceptions of COVID-19 vaccine development timelines in Canada and USA
Source: PLoS One. 2022 Feb 15;17(2):e0262740. doi: 10.1371/journal.pone.0262740 (PMC8846503; doi:10.1371/journal.pone.0262740)
Supplement: S1 Appendix — (DOCX) [file pone.0262740.s001.docx]

**Supplemental material for Expert Forecasts of COVID-19 Vaccine Development Timelines**

Patrick Bodilly Kane, Brian Ward, Jesse Papenburg, Stephen B. Broomell, Hannah Moyer, Amanda MacPherson1, Jonathan Kimmleman

**Expert Characteristics**

Nineteen of our experts agreed to have their names included in the manuscript. Their names and affiliations are found in Table S1. The median age of our experts was 59 (range 35-80); 8 were women. One expert, identified as having 0 years of vaccine development experience, but reported 30 years of experience with vaccine usage in transplants, we recorded this expert as having 30 years of experience when calculating experience.

| Name | Affiliation |
| --- | --- |
| Luis Barreto | Formerly Sanofi |
| Dawn Bowdish | McMaster University |
| Bruce Forrest | Formerly Wyeth |
| Eduardo Gotuzzo | Cayetano Heredia University, Lima, Peru |
| Scott Halperin | Dalhousie University |
| Shehzad Iqbal | GlaxoSmithKline (GSK) |
| Anthony Jevnikar | University of Western Ontario |
| Jim Kellner | University of Calgary |
| Gary Kobinger | Laval University |
| Jeff Kwong | University of Toronto |
| Michael Libman | McGill University |
| Mark Loeb | McMaster University |
| Matthew Miller | McMaster University |
| Monika Naus | British Columbia Centres for Disease Control (BC-CDC) |
| David Scheifele | University of British Columbia |
| Andy sheldon | IVM vaccines |
| Mali Stephanie | Federal Agency for Medicines and Health Products |
| Tania Watts | University of Toronto |

**Table S1 –** Names and affiliations for experts who agreed to have their identities made public.

**Expert Sample Creation:**

BJW composed a list of experts that he felt comfortable contacting directly that spanned industry, academia and public health and represented a diverse range of viewpoints and backgrounds.

This list of experts was supplemented with corresponding author searches for vaccine review articles from the following journals: *NEJM,* *JAMA*, *Lancet*, *Annals Int. Med*, *Nature, Immunology*, *Immunity*, *Cell Host and Microbe*, *Journal of Experimental Medicine*, *Science Immunology*, *PLoS Pathogens or Medicine*, *PLoS Neg. Tropical Disease*, *PNAS*, *J Clinical, Investigation*, *Nature Reviews Immunology*, *Annual Reviews of Immunology*, *Trends in, Immunology*, *Immunological Reviews*, *Vaccines*, *Journal of Inf. Disease*.

**Survey Design**

The survey was designed using an iterative process in which BJW and JP were queried on important stages of vaccine development and how candidates might fail. Milestones and potential setback questions were drafted based on these ideas. BJW (the author with the most vaccine development expertise) was then presented with the drafts to provide comments. Based on the comments the questions were further refined. There were several constraints on this process. First, we aimed to get questions to which experts could provide meaningful and informative answers. Second, we aimed for questions for which it would be relatively straightforward to verify if they had in fact occurred. Finally, we planned to ask the same questions to lay people, so we aimed for milestones that could be understood by a lay audience with minimal additional text.

The survey was distributed to four vaccine or infectious disease experts within the contact circle of co-authors for piloting to test question clarity and survey length. Minor revisions were made based on their feedback.

The final text for the three milestone items is as follows:

1. When do you think a field trial enrolling more than 5000 participants will report final results testing a COVID-19 vaccine candidate?
2. When do you think a COVID-19 vaccine will be available in the US and/or Canada for those at the highest risk of contracting the virus, like health care workers, including availability under emergency use authorization?
3. When do you think a COVID-19 vaccine will be readily available for the general public (over the age of 18) in the US and/or Canada?

The final text for the two setback questions is as follows:

1. What is the probability that the first COVID-19 vaccine to be widely deployed in the US and/or Canada receives a black box warning from the FDA (the FDA issues black box warnings when a safety issue is detected after a vaccine has been approved)?
2. What is the probability that the first large field trial in the US and/or Canada of a COVID-19 vaccine has a negative or null efficacy outcome?

The lay version of the survey used slightly different text for the milestones:

1. When do you think a field trial enrolling more than 5000 participants will report final results testing a COVID-19 vaccine candidate?
2. When do you think a COVID-19 vaccine will be available in the US and/or Canada for those at the highest risk of contracting the virus, like health care workers?
3. When do you think a COVID-19 vaccine will be readily available for the general public (over the age of 18) in the US and/or Canada?

And for the setback questions:

1. Sometimes after a vaccine has been approved by the FDA it will be discovered that it has some safety issues that were not discovered in testing.  When this happens the FDA will often issue a warning about the vaccine, referred to as a black box warning. What is the probability that the first COVID-19 vaccine to be widely deployed in the US and/or Canada will have safety issues that require the FDA to issue a black box warning?
2. Sometimes vaccine candidates that show promise in early human trials, are later found to be ineffective in more rigorous field trials. What is the probability that the first large field trial in the US and/or Canada of a COVID-19 vaccine finds that the vaccine is ineffective at preventing infection?

The current manuscript is part of a larger study that was preregistered at OSF. You can view the preregistration here: <https://osf.io/faz9q>.

**Survey Interface**

**Data Cleaning**

Two experts indicated that they were somewhat uncomfortable with answering questions about the US and Canadian health care system so we removed their responses to the milestone questions about vaccine availability in the US and/or Canada, and for the setback question about an FDA black box warning. This had no effect on median estimates.

Experts could indicate that they believed a milestone would not occur in the next 10 years. For the purposes of calculating medians and interquartile ranges we treated this answer as though it was an answer of 11 years. Due to the nature of medians and interquartile ranges the choice of 11 years as opposed to a longer time frame had no impact on the data. For both the milestones about vaccine availability 1 expert indicated they thought the latest the milestone could occur was not in the next 10 years. Finally no experts provided only year information without month information.

**Milestone Plots**

Plots of the individual level forecasts for each milestone are contained in Figures S1, S2 and S3. Note that expert number 9 and expert number 15 indicated some level of discomfort with the US and Canadian healthcare systems.

**Setback Questions**

Figure S4 contains histograms of experts predicted probabilities of the FDA issuing a blackbox warning on an approved vaccine and the first large field study reporting a null or negative outcome on a primary efficacy endpoint.


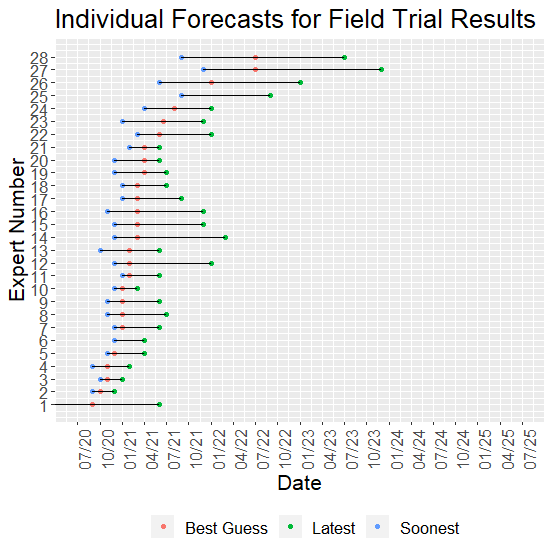


**Figure S1** – Individual forecasts for the field trial results milestone.


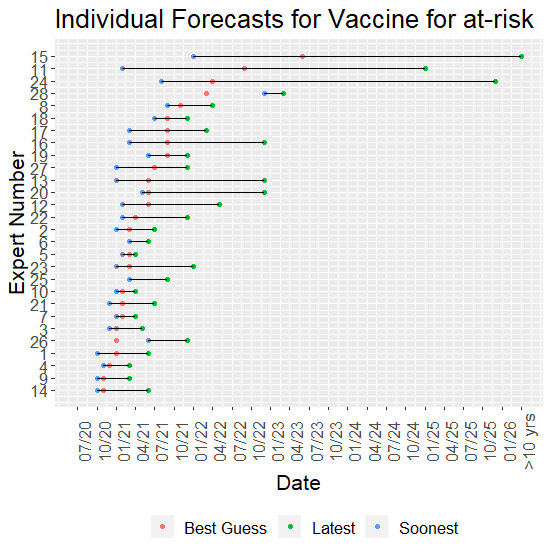


**Figure S2** – Individual forecasts for the vaccine available to at-risk individuals milestone. The numbering of the experts is the same as in Figure A3. Note the break in the x-axis.


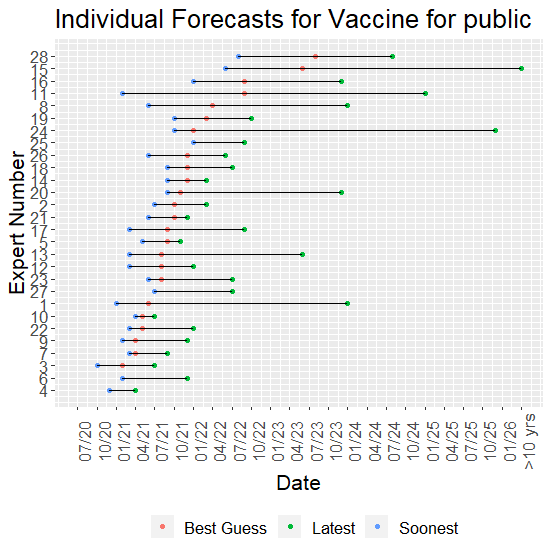


**Figure S3** – Individual forecasts for the vaccine available to the public milestone. The numbering of the experts is the same as in Figure A3. Note the break in the x-axis.


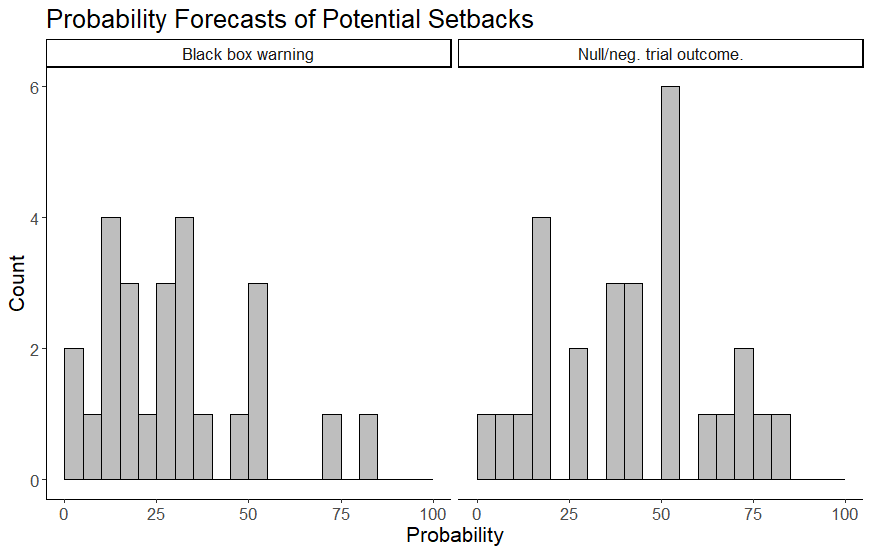


**Figure S4 –** Histograms of probabilities provided for the two setback questions.

**Inconsistent and Missing Responses**

The percentage of lay responses that were inconsistent either because the best estimate was not between the soonest and latest estimates or because the participant had indicated that the milestone had not been reached but nevertheless provided an answer in the past. Additionally, in the US sample there were 168 and 136 missing responses for the probability of a boxed warning and probability of the first large trial returning a null outcome, respectively. For the Canadian sample there were 239 and 198 for the same. Table S2 contains complete information on missing responses.

| **US** | **Best Guess Before Soonest, After Latest** | **Response in Past** | **Total Inconsistent Responses** | **Indicated Milestone Already Occurred** | **Missing Response** |
| --- | --- | --- | --- | --- | --- |
| Field Study Results | 191 (19%) | 16 (2%) | 207 (20%) | 131 (13%) | 6 (1%) |
| Vaccine for at-risk | 235 (23%) | 18 (2%) | 253 (25%) | 45 (4%) | 13 (2%) |
| Vaccine for Public | 259 (25%) | 10 (1%) | 269 (27%) | 45 (4%) | 14 (2%) |
| **Canada** |  |  |  |  |  |
| Field Study Results | 137 (13%) | 8 (1%) | 145 (14%) | 61 (6%) | 4 (1%) |
| Vaccine for at-risk | 142 (13%) | 10 (1%) | 152 (14%) | 22 (2%) | 17 (2%) |
| Vaccine for Public | 158 (15%) | 11 (1%) | 169 (16%) | 22 (2%) | 13 (2%) |

**Table S2** – Number and percentage of sample by reasons for exclusion of lay responses in analysis. Also includes those who indicated they believed the milestone had already occurred.

**Soonest Estimate Latest Estimate Ranges**

In addition to testing for differences in best estimates between the three samples we also tested for differences in the median range between soonest and latest estimates. The median ranges and p-values for comparisons between the three samples can be found in Table S3. Across all three milestones experts tended to provide narrower ranges between their soonest and latest guesses than either group of lay people. Lay people in the US and Canada provided ranges that were approximately the same.

|  | **Range** | | | **P-value** | | |
| --- | --- | --- | --- | --- | --- | --- |
| **Question** | **Expert** | **US** | **Canada** | **Expert vs US** | **Expert vs Canada** | **US vs Canada** |
| **Field Study Results** | 8 | 12 | 12 | <0.001* | <0.001* | 0.90 |
| **Vaccine For At-Risk** | 7 | 12 | 12 | <0.001* | <0.001* | 0.99 |
| **Vaccine For Public** | 11 | 12 | 12 | <0.001* | <0.001* | 0.99 |

**Table S3** – Median range between soonest and latest estimates for each milestone for the expert, US lay and Canadian lay samples. P-values are from bootstrapped significance tests testing whether the median ranges are different between samples.

**Scale Questions**

We asked participants in the lay survey a series of five questions which we intended to combine into a scale to measure their perceptions of how the accelerated pace of COVID research would affect the prevalence of errors in research. Participants rated their agreement with the following five statements using a Likert scale ranging from Strongly Disagree to Strongly Agree:

1.The increased urgency associated with COVID-19 research will lead to more errors in the research and development process.

2.Expert opinions about COVID-19 are generally trustworthy.

3.The scientific community has made a lot of mistakes when dealing with COVID-19.

4.COVID-19 research oversight and error monitoring should be relaxed because of the need for treatments.

5.It is unlikely that COVID-19 research suffers from any major errors.

Questions 1 and 3 were reverse coded. The correlation matrix for the scale can be found in Table S4. Overall we found the scale was unreliable with a Cronbach’s alpha of 0.43. We were additionally unable to find a subset of the items with a Cronbach’s alpha greater than 0.6 so in accordance with our preregistration we dropped all items except for Question 1 from further analysis.

|  | **Q1** | **Q2** | **Q3** | **Q4** | **Q5** |
| --- | --- | --- | --- | --- | --- |
| **Q1** | 1.00 |  |  |  |  |
| **Q2** | -0.01 | 1.00 |  |  |  |
| **Q3** | 0.38 | -0.23 | 1.00 |  |  |
| **Q4** | 0.11 | 0.12 | 0.23 | 1.00 |  |
| **Q5** | -0.06 | 0.30 | 0.00 | 0.41 | 1.00 |

**Table S4** – Correlation matrix for the questions measuring belief that increased pace of COVID-19 research would lead to additional errors.

**Regressors**

Distributions for how far individuals believed a vaccine had already progressed through the approval process (a categorial variable with levels for different stages reached by the most advanced vaccine) and agreement with the statement “The increased urgency associated with COVID-19 research will lead to more errors in the research and development process” on a 5-point Likert scale are found in Table S5. The variables are used as regressors in our exploratory analysis.

|  | **US** | **Canada** |
| --- | --- | --- |
| **How far a vaccine already progressed through the approval process?** |  |  |
| Pre-clinical | 202 | 259 |
| Phase 1 | 262 | 311 |
| Phase 2 | 266 | 294 |
| Phase 3 | 118 | 70 |
| Approval | 86 | 30 |
| Manufacture and Distribution | 45 | 23 |
| **The increased urgency associated with COVID-19 research will lead to more errors in the research and development process** |  |  |
| Strongly Disagree | 36 | 21 |
| Disagree | 104 | 160 |
| Neither Agree nor Disagree | 294 | 282 |
| Agree | 405 | 484 |
| Strongly Agree | 175 | 107 |

**Table S5** – Summary of response to “How far a vaccine already progressed through the approval process?” and agreement with “The increased urgency associated with COVID-19 research will lead to more errors in the research and development process”.

**Regression Coefficients**

For each milestone best estimate and setback question, we subtracted the expert median from each lay response to create a set of deviation variables. The distribution of each deviation variable are plotted in figures S5 and S6. F-tests comparing the full regression model with models without each individual regressor are included in Table A6 along with effect sizes. Coefficients and standard errors for the five regression we fit are included in Table A7. The reference levels for education, gender, ethnicity and nationality are a high school diploma, female, white and Canadian.


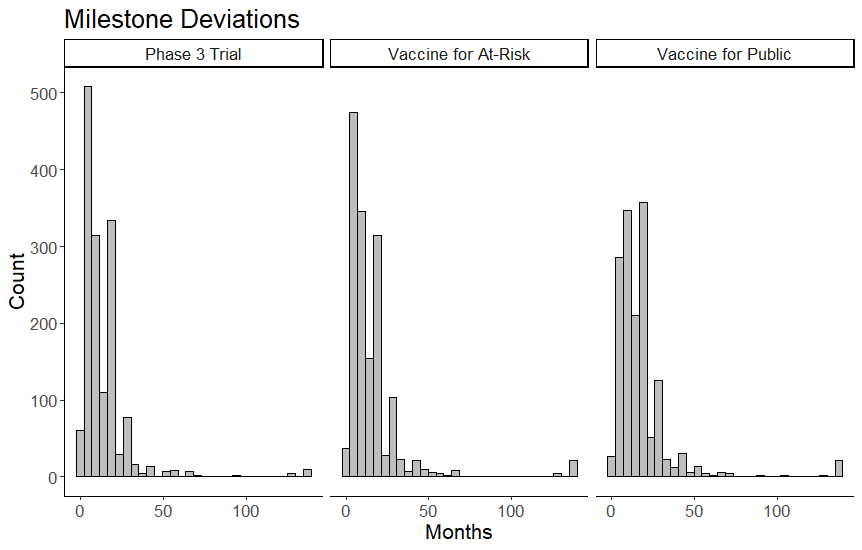


**Figure S5** – Distribution of deviation variables for the Phase 3 trial, vaccine available to most at-risk individuals and vaccine available to the public milestones.


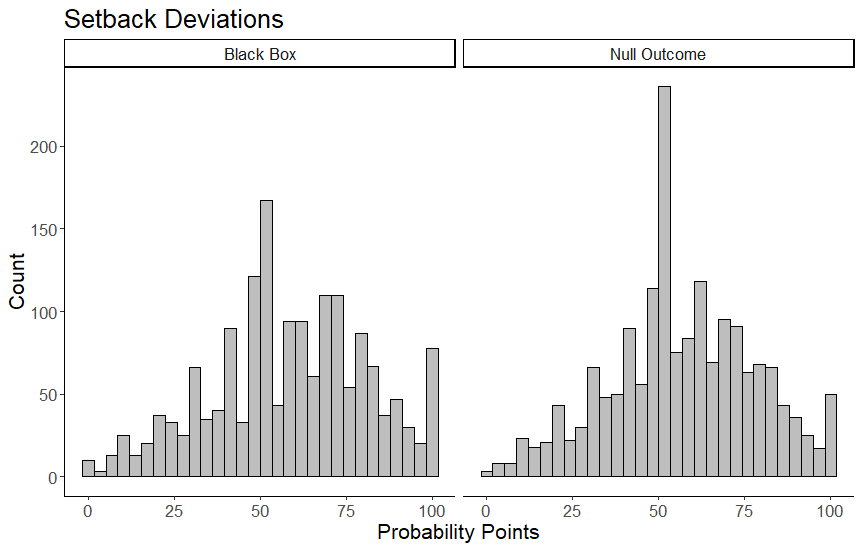


**Figure S6** – Distribution of deviation variables for the boxed warning and the null outcome setbacks.

|  | **Variable** | **F** | **DFs** | **P-value** | **η^2^** |
| --- | --- | --- | --- | --- | --- |
| **Best Estimate of Field Study Result (R^2^=0.03)** | Education | 2.27 | (6, 1389) | 0.03* | 0.0098 |
|  | Gender | 1.2 | (4, 1389) | 0.3 | 0.0034 |
|  | Age | 0.94 | (2, 1389) | 0.4 | 0.0013 |
|  | Ethnicity | 2.05 | (8, 1389) | 0.04* | 0.012 |
|  | Political | 1.24 | (2, 1389) | 0.28 | 0.0018 |
|  | Belief about Stage of Approval Process | 4.71 | (2, 1389) | <0.01* | 0.013 |
|  | Pace of Research Causes Errors | 2.36 | (2, 1389) | 0.09 | 0.0034 |
|  | Nationality Interaction | 1.03 | (14, 1389) | 0.41 | 0.01 |
| **Best Estimate of Vaccine For At-Risk (R^2^=0.03)** | Education | 1.84 | (6, 1452) | 0.09 | 0.0075 |
|  | Gender | 1.63 | (4, 1452) | 0.16 | 0.0045 |
|  | Age | 0.92 | (2, 1452) | 0.4 | 0.0013 |
|  | Ethnicity | 1.24 | (8, 1452) | 0.27 | 0.0068 |
|  | Political | 0.28 | (2, 1452) | 0.74 | 0.004 |
|  | Belief about Stage of Approval Process | 4.73 | (2, 1452) | <0.01* | 0.013 |
|  | Pace of Research Causes Errors | 2.03 | (2, 1452) | 0.13 | 0.0028 |
|  | Nationality Interaction | 1.58 | (14, 1452) | 0.07 | 0.015 |
| **Best Estimate of Vaccine For Public (R^2^=0.09)** | Education | 1.1 | (6, 1429) | 0.37 | 0.0046 |
|  | Gender | 1.85 | (4, 1429) | 0.11 | 0.0052 |
|  | Age | 0.26 | (2, 1429) | 0.77 | 0.0004 |
|  | Ethnicity | 1.35 | (8, 1429) | 0.21 | 0.0075 |
|  | Political | 0.52 | (2, 1429) | 0.59 | 0.0007 |
|  | Belief about Stage of Approval Process | 4.98 | (2, 1429) | <0.01* | 0.014 |
|  | Pace of Research Causes Errors | 5.12 | (2, 1429) | <0.01* | 0.0071 |
|  | Nationality Interaction | 1.59 | (14, 1429) | 0.07 | 0.015 |
| **Probability of Black Box (R^2^=0.09)** | Education | 1.55 | (6, 1542) | 0.16 | 0.006 |
|  | Gender | 4.84 | (4, 1542) | <0.01* | 0.012 |
|  | Age | 0.05 | (2, 1542) | 0.94 | 0.0001 |
|  | Ethnicity | 0.51 | (8, 1542) | 0.84 | 0.0027 |
|  | Political | 2.17 | (2, 1542) | 0.11 | 0.0028 |
|  | Belief about Stage of Approval Process | 1.14 | (2, 1542) | 0.34 | 0.003 |
|  | Pace of Research Causes Errors | 68.86 | (2, 1542) | <0.01* | 0.082 |
|  | Nationality Interaction | 1.31 | (14, 1542) | 0.17 | 0.012 |
| **Probability of Null Outcome (R^2^=0.10)** | Education | 0.95 | (6, 1542) | 0.46 | 0.0035 |
|  | Gender | 0.98 | (4, 1542) | 0.42 | 0.0024 |
|  | Age | 0.87 | (2, 1542) | 0.42 | 0.0011 |
|  | Ethnicity | 0.91 | (8, 1542) | 0.51 | 0.0045 |
|  | Political | 0.95 | (2, 1542) | 0.39 | 0.0012 |
|  | Belief about Stage of Approval Process | 0.75 | (2, 1542) | 0.56 | 0.0019 |
|  | Pace of Research Causes Errors | 83.81 | (2, 1542) | <0.01* | 0.094 |
|  | Nationality Interaction | 0.92 | (14, 1542) | 0.54 | 0.0079 |

**Table S6** – Results of F-tests for nested model comparisons testing whether each individual predictor significantly improved the fit of the models, along with the effect sizes on these tests (**η^2^**). Critical values were not adjusted for multiple hypothesis testing. R^2^ refers to the variance explained by the full regression.

|  | **Field Study Results** | | **Vaccine for At-risk** | | **Vaccine for Public** | | **Boxed Warning** | | **Null Outcome** | |
| --- | --- | --- | --- | --- | --- | --- | --- | --- | --- | --- |
| **Variable** | b | SE | b | SE | b | SE | b | SE | b | SE |
| **Intercept** | 12.61 | 3.81 | 12.75 | 4.38 | 7.80 | 4.51 | 10.90 | 5.01 | -5.74 | 4.60 |
| **The Pace of Research Leads to More Errors** | 0.98 | 0.64 | 0.853 | 0.735 | 1.64 | 0.75 | 7.45 | 085 | 6.34 | 0.77 |
| **University** | 0.31 | 1.39 | 0.44 | 1.58 | -0.18 | 1.62 | 1.59 | 1.88 | -0.34 | 1.69 |
| **Graduate Degree** | 2.71 | 1.93 | 4.41 | 2.22 | 3.42 | 2.27 | -3.12 | 2.47 | 0.58 | 2.26 |
| **Other Degree** | 0.09 | 1.97 | 1.94 | 2.21 | 0.56 | 2.29 | -1.24 | 2.70 | -1.58 | 2.52 |
| **Age** | -0.02 | 0.04 | 0.00 | 0.05 | 0.00 | 0.05 | 0.00 | 0.05 | 0.04 | 0.05 |
| **Male** | -2.70 | 1.25 | -3.25 | 1.39 | -3.83 | 1.23 | -5.89 | 1.61 | 0.07 | 1.47 |
| **Other Gender** | -2.65 | 8.36 | 0.57 | 9.60 | -2.07 | 9.83 | -49.90 | 21.81 | -20.93 | 20.36 |
| **Political Stance** | -0.71 | 0.45 | -0.24 | 0.51 | -0.12 | 0.53 | -1.13 | 0.59 | 0.03 | 0.53 |
| **Belief about Stage in the Approval Process** | -2.20 | 0.65 | -2.70 | 0.65 | -2.96 | 0.68 | -0.66 | 0.73 | -0.64 | 0.67 |
| **Asian** | -3.48 | 1.58 | -3.12 | 1.80 | -3/05 | 1.84 | -2.43 | 2.00 | -0.39 | 1.83 |
| **Black** | 0.14 | 4.23 | -2.78 | 4.38 | -4.74 | 4.71 | -2.07 | 4.98 | -7.14 | 4.35 |
| **Latinx** | 11.12 | 5.33 | 9.38 | 5.86 | 14.21 | 6.58 | 5.37 | 6.67 | 3.37 | 5.97 |
| **Other Ethnicity** | -1.37 | 2.26 | -1.33 | 2.57 | -0.10 | 2.60 | 3.28 | 3.22 | 2.68 | 2.99 |
| **US** | -2.73 | 5.53 | -4.64 | 6.25 | -5.46 | 6.43 | -0.01 | 6.82 | 2.31 | 6.23 |
| **The Pace of Research Leads to More Errors*US** | 0.05 | 0.93 | 0.39 | 1.05 | 0.16 | 1.08 | -1.51 | 1.14 | 0.58 | 1.04 |
| **University*US** | -5.38 | 2.06 | -4.43 | 2.29 | -2.34 | 2.36 | -065 | 2.59 | 1.90 | 2.36 |
| **Graduate Degree*US** | -4.11 | 2.75 | -4.53 | 3.11 | -7.15 | 3.23 | 7.63 | 3.23 | 3.63 | 2.96 |
| **Other Degree*US** | -5.38 | 4.19 | -5.16 | 4.90 | -3.03 | 5.02 | -1.23 | 5.68 | 0.58 | 4.96 |
| **Age*US** | -0.03 | 0.06 | -0.07 | 0.07 | -0.03 | 0.07 | 0.02 | 0.08 | -0.09 | 0.07 |
| **Male*US** | 2.34 | 1.86 | 1.72 | 2.06 | 3.11 | 2.12 | 4.26 | 2.27 | -2.07 | 2.08 |
| **Other Gender*US** | 3.23 | 12.70 | 0.80 | 13.57 | 2.74 | 13.88 | 42.68 | 23.89 | 10.96 | 22.30 |
| **Political Stance*US** | 0.70 | 0.61 | -0.05 | 0.69 | -0.36 | 0.72 | 0.74 | 0.75 | -0.62 | 0.69 |
| **Belief about Stage in the Approval Process*US** | 0.61 | 0.94 | 2.63 | 0.90 | 2.50 | 0.93 | -0.40 | 0.92 | 0.32 | 0.84 |
| **Asian*US** | 4.83 | 3.81 | 6.36 | 4.13 | -0.12 | 4.26 | 3.86 | 4.14 | 5.05 | 3.81 |
| **Black*US** | 2.91 | 4.68 | 5.25 | 4.93 | 5.85 | 5.23 | 2.23 | 5.46 | 6.18 | 4.82 |
| **Latinx*US** | -5.72 | 6.13 | -9.05 | 6.67 | -12.66 | 7.38 | -4.89 | 7.37 | -3.67 | 6.62 |
| **Other Ethnicity*US** | 5.78 | 3.65 | 6.18 | 4.07 | 3.29 | 4.18 | -2.42 | 4.66 | -5.14 | 4.31 |

**Table S7** – Coefficients and standard errors of all regressions.
