# Supplementary material for: Relationship between lay and expert perceptions of COVID-19 vaccine development timelines in Canada and USA
Source: PLoS One. 2022 Feb 15;17(2):e0262740. doi: 10.1371/journal.pone.0262740 (PMC8846503; doi:10.1371/journal.pone.0262740)
Supplement: S1 File — (ZIP) [file pone.0262740.s002.zip › Data/Codebook.docx]

Datasets:

US_vaccine.csv – A Comma Separated Values file containing data for the US sample.

Canada_vaccine.csv – A Comma Separated Values file containing data for the English speaking Canadian sample.

French_vaccine.csv - A Comma Separated Values file containing data for the French speaking Canadian sample.

Variables:

P3.best – Participants’ best estimate of when a field trial enrolling more than 5000 participants will report final results testing a COVID-19 vaccine candidate would occur. Participants could give answers between January 2020 and December 2030 and could also indicate that it would take even longer than that. Coded as months since July 2020. A value of 0 indicates a response of July 2020 and negative values indicate responses prior to July 2020. Responses of longer than 10 years were coded as the equivalent of July 2031.

P3.soonest – The soonest participants thought a field trial enrolling more than 5000 participants will report final results testing a COVID-19 vaccine candidate would occur. Participants could give answers between January 2020 and December 2030 and could also indicate that it would take even longer than that. Coded as months since July 2020. A value of 0 indicates a response of July 2020 and negative values indicate responses prior to July 2020. Responses of longer than 10 years were coded as the equivalent of July 2031.

P3.latest – The latest participants thought a field trial enrolling more than 5000 participants will report final results testing a COVID-19 vaccine candidate would occur. Participants could give answers between January 2020 and December 2030 and could also indicate that it would take even longer than that. Coded as months since July 2020. A value of 0 indicates a response of July 2020 and negative values indicate responses prior to July 2020. Responses of longer than 10 years were coded as the equivalent of July 2031.

atrisk.best – Participants’ best estimate of when a vaccine would be available to those most at risk. Participants could give answers between January 2020 and December 2030 and could also indicate that it would take even longer than that. Coded as months since July 2020. A value of 0 indicates a response of July 2020 and negative values indicate responses prior to July 2020. Responses of longer than 10 years were coded as the equivalent of July 2031.

atrisk.soonest – The soonest participants thought a vaccine would be available to those most at risk. Participants could give answers between January 2020 and December 2030 and could also indicate that it would take even longer than that. Coded as months since July 2020. A value of 0 indicates a response of July 2020 and negative values indicate responses prior to July 2020. Responses of longer than 10 years were coded as the equivalent of July 2031.

atrisk.latest – The latest participants thought a vaccine would be available to those most at risk. Participants could give answers between January 2020 and December 2030 and could also indicate that it would take even longer than that. Coded as months since July 2020. A value of 0 indicates a response of July 2020 and negative values indicate responses prior to July 2020. Responses of longer than 10 years were coded as the equivalent of July 2031.

atrisk.best – Participants’ best estimate of when a vaccine would be available to the public. Participants could give answers between January 2020 and December 2030 and could also indicate that it would take even longer than that. Coded as months since July 2020. A value of 0 indicates a response of July 2020 and negative values indicate responses prior to July 2020. Responses of longer than 10 years were coded as the equivalent of July 2031.

atrisk.soonest – The soonest participants thought a vaccine would be available to the public. Participants could give answers between January 2020 and December 2030 and could also indicate that it would take even longer than that. Coded as months since July 2020. A value of 0 indicates a response of July 2020 and negative values indicate responses prior to July 2020. Responses of longer than 10 years were coded as the equivalent of July 2031.

atrisk.latest – The latest participants thought a vaccine would be available to the public. Participants could give answers between January 2020 and December 2030 and could also indicate that it would take even longer than that. Coded as months since July 2020. A value of 0 indicates a response of July 2020 and negative values indicate responses prior to July 2020. Responses of longer than 10 years were coded as the equivalent of July 2031.

Blackbox – Participants’ estimate of the probability a COVID-19 vaccine would receive a black box warning from the FDA.

Nulloutcome – Participants’ estimate of the probability a COVID-19 vaccine trial would result in a null or negative outcome.

q1 – Participant agreement with the statement: “The increased urgency associated with COVID-19 research will lead to more errors in the research and development process” on a five-point Likert scale ranging from “Strongly disagree” to “Strongly agree.

q2 – Participant agreement with the statement: “Expert opinions about COVID-19 are generally trustworthy” on a five-point Likert scale ranging from “Strongly disagree” to “Strongly agree.

q3 – Participant agreement with the statement: “The scientific community has made a lot of mistakes when dealing with COVID-19” on a five-point Likert scale ranging from “Strongly disagree” to “Strongly agree.

q4 – Participant agreement with the statement: “COVID-19 research oversight and error monitoring should be relaxed because of the need for treatments” on a five-point Likert scale ranging from “Strongly disagree” to “Strongly agree.

q5 – Participant agreement with the statement: “It is unlikely that COVID-19 research suffers from any major errors” on a five-point Likert scale ranging from “Strongly disagree” to “Strongly agree.

education – Highest education achieved with: 1=High School, 2=Some University, 3=University, 4=Master’s, 5=MD, 6=PhD, 7=MD/PhD and 8=Other with the option to specify.

age – Age in years.

gender – Gender with: 1=Female, 2=Male, 3=Non-binary, 4=Prefer to specify with textual entry and 5=Prefer not to answer. Participants could select more than 1.

ethnicity – Ethnicity with 1=Asian, 2=Black or African American, 3=Hispanic or Latinx, 4=Middle Eastern or North African, 5=Native American or Indigenous, 6=Pacific Islander, 7=White, 8=Some unlisted group with textual entry and 9=Prefer not to answer. Participants could select more than one answer.

stance – Self described political stance on a scale from 1 being “Far Right” to 7 being “Far Left” with “Center” as the midpoint.

party – Self described political party. For the US survey answers could be: 1=Democratic Party, 2=Independent, 3=Republican and 4=Other with the option to specify. For the Canadian survey the answers could be: 1=Liberal, 2=Conservative, 3=NDP, 4=Green, 5=Block Quebecois, 6=Independent and 7=Other with the option to specify.

Knowledge – How far participants believe vaccine development had already progressed at the time of the survey. Answers included: 1=Pre-clinical research, 2=Phase I, 3=Phase II, 4=Phase III, 5=Approval by FDA and 6=Manufacture and Distribution
